# ABT-888 restores sensitivity in temozolomide resistant glioma cells and xenografts

**DOI:** 10.1371/journal.pone.0202860

**Published:** 2018-08-28

**Authors:** Alice L. Yuan, Christian B. Ricks, Alexandra K. Bohm, Xueqing Lun, Lori Maxwell, Shahana Safdar, Shazreh Bukhari, Amanda Gerber, Wajid Sayeed, Elizabeth. A. Bering, Haley Pedersen, Jennifer A. Chan, Yaoqing Shen, Marco Marra, David R. Kaplan, Warren Mason, Lindsey D. Goodman, Ravesanker Ezhilarasan, Ascher B. Kaufmann, Matthew Cabral, Steve M. Robbins, Donna L. Senger, Daniel P. Cahill, Erik P. Sulman, J. Gregory Cairncross, Michael D. Blough

**Affiliations:** 1 Clark H. Smith Brain Tumour Centre, University of Calgary, Calgary, Alberta, Canada; 2 Charbonneau Cancer Institute, University of Calgary, Calgary, Alberta, Canada; 3 Department of Radiation Oncology, MD Anderson Cancer Center, Houston, Texas, United States of America; 4 Department of Oncology, University of Calgary, Calgary, Alberta, Canada; 5 Department of Pathology & Laboratory Medicine, University of Calgary, Calgary, Alberta, Canada; 6 Michael Smith Genome Sciences Centre, British Columbia Cancer Agency, Vancouver, British Columbia, Canada; 7 Department of Molecular Genetics, University of Toronto, Toronto, Ontario, Canada; 8 Program in Neurosciences and Mental Health, Hospital for Sick Children, Toronto, Ontario, Canada; 9 Princess Margaret Cancer Centre, Toronto, Ontario, Canada; 10 Department of Neurosurgery, MD Anderson Cancer Center, Houston, Texas, United States of America; 11 Department of Neurosurgery, Massachusetts General Hospital, Boston, Massachusetts, United States of America; 12 Department of Clinical Neurosciences, University of Calgary, Calgary, Alberta, Canada; 13 Hotchkiss Brain Institute, University of Calgary, Calgary, Alberta, Canada; Northern University, UNITED STATES

## Abstract

**Background:**

Temozolomide (TMZ) is active against glioblastomas (GBM) in which the O6-methylguanine-DNA methyltransferase (*MGMT*) gene is silenced. However, even in responsive cases, its beneficial effect is undermined by the emergence of drug resistance. Here, we tested whether inhibition of poly (ADP-ribose) polymerase-1 and -2 (PARP) enhanced the effectiveness of TMZ.

**Methods:**

Using patient derived brain tumor initiating cells (BTICs) and orthotopic xenografts as models of newly diagnosed and recurrent high-grade glioma, we assessed the effects of TMZ, ABT-888, and the combination of TMZ and ABT-888 on the viability of BTICs and survival of tumor-bearing mice. We also studied DNA damage repair, checkpoint protein phosphorylation, and DNA replication in mismatch repair (MMR) deficient cells treated with TMZ and TMZ plus ABT-888.

**Results:**

Cells and xenografts derived from newly diagnosed *MGMT* methylated high-grade gliomas were sensitive to TMZ while those derived from unmethylated and recurrent gliomas were typically resistant. ABT-888 had no effect on the viability of BTICs or tumor bearing mice, but co-treatment with TMZ restored sensitivity in resistant cells and xenografts from newly diagnosed unmethylated gliomas and recurrent gliomas with *MSH6* mutations. In contrast, the addition of ABT-888 to TMZ had little sensitizing effect on cells and xenografts derived from newly diagnosed methylated gliomas. In a model of acquired TMZ resistance mediated by loss of MMR gene *MSH6*, re-sensitization to TMZ by ABT-888 was accompanied by persistent DNA strand breaks, re-engagement of checkpoint kinase signaling, and interruption of DNA synthesis.

**Conclusion:**

In laboratory models, the addition of ABT-888 to TMZ overcame resistance to TMZ.

## Introduction

High-grade gliomas are aggressive cancers for which there is no curative treatment. For GBM, the standard of care includes the alkylating agent TMZ, and for oligodendroglioma, best practice includes lomustine, another alkylating agent [1, 2]. For both, anti-tumor action is primarily mediated by toxic methyl adducts at O6-guanine. Subsequent mispairing of nucleotides during DNA replication and futile activation of the mismatch repair (MMR) system leads to lethal double-strand breaks [3, 4]. Although TMZ and Lomustine are effective when given together with radiation (RT), many newly diagnosed high-grade gliomas, especially GBMs, are intrinsically resistant to alkylating agents, while others respond initially, but acquire resistance [2]. Curative therapies for GBM seem distant, but the ability to enhance existing treatments may be possible, and the focus of this report.

The primary mechanism of intrinsic resistance to TMZ and CCNU is removal of methyl adducts from O6-guanine by O6-methylguanine-DNA methyltransferase (MGMT) [5]. In the clinic, MGMT repair of therapeutic DNA damage induced by TMZ and CCNU is associated with shorter median survival times and lower two and five-year survival rates in patients receiving chemotherapy and RT. Indeed, patients whose tumors do not express MGMT live significantly longer [6–8]. Other DNA repair mechanisms have also been associated with resistance to alkylating agents in glioma patients, notably base-excision repair (BER) and homologous recombination (HR) [9–11].

One strategy to overcome resistance mediated by BER and HR is to block DNA repair by inhibiting poly (ADP-ribose) polymerase-1 and -2 (collectively referred to as PARP), DNA damage sensors that bind to single and double-strand DNA breaks to catalyze the synthesis of ADP-ribose polymers [12–15]. These polymers serve as scaffolds to recruit and modify the function of downstream DNA repair proteins, activating the BER and HR pathways [15]. As such, inhibition of PARP interferes with DNA repair and has the potential to increase the efficacy of cancer treatments that incorporate alkylating agents.

PARP inhibition has been shown to potentiate TMZ in pre-clinical models of several cancers [16–18]. The effects of PARP inhibition on TMZ sensitivity have also been tested in glioma models, including conventional and primary cell lines and xenografts [19–25], BTICs and xenografts [19, 21, 23], and syngeneic and genetically modified animal models [16, 26]. The results of these studies have been inconsistent, providing no clear guidance for clinicians. In some lines and xenografts, inhibition of PARP potentiates sensitivity to TMZ, while in others there is no observable benefit [24, 25]. Furthermore, several studies have found PARP inhibition potentiates TMZ only in TMZ-sensitive tumors [24, 25], whereas others have noted potentiation in the setting of TMZ resistance [19–23, 26]. Because there are many possible explanations for such discrepancies, we decided to re-evaluate the role of PARP inhibition in the treatment of gliomas using multiple BTIC lines derived from newly diagnosed and recurrent GBMs and oligodendrogliomas and in orthotopic xenografts. Using clinically relevant drug doses and schedules, and cells and xenograft models from patients with known *MGMT* status [27, 28], we found that ABT-888 enhanced sensitivity to TMZ and that this beneficial effect was most apparent in cells and xenografts derived from resistant tumours, confirming an observation made previously by other groups [21, 29]. We used an isogenic BTIC system to explore the mechanistic basis of this phenomenon.

## Materials and methods

### Cell culture

BTICs (n = 15) were derived from newly diagnosed and recurrent high-grade gliomas were obtained from the laboratory of Dr. Samuel Weiss at the University of Calgary during the period between 2008 and 2012. All BTIC lines were used within 25–30 passages of line establishment from primary cells. In brief, following informed consent from GBM patients, GBM BTICs were cultured from tumor specimens obtained during operative procedures as previously described [27] and approved by the University of Calgary Ethics Review Board and the Health Research Ethics board of Alberta—Cancer Commitee (HREBA, Protocol **#** HREBA.CC-16-0153). Newly diagnosed lines were derived from treatment-naïve patients and recurrent ones from patients who had been treated previously with TMZ and RT. BTICs were maintained at 37°C in a humidified 5% CO_2_ environment and grown in serum-free NeuroCult^™^ media (Stem Cell Technologies) supplemented with 0.1% heparin (Stem Cell Technologies), 0.1% rhesus fibroblast growth factor and 0.1% rhesus epidermal growth factor (Peprotech). Pyrosequencing to assess *MGMT* methylation status was performed by EpigenDx (Orlando, FL) using protocol ADS1552.

### In vitro drug testing and cell viability

**Fig 1:**
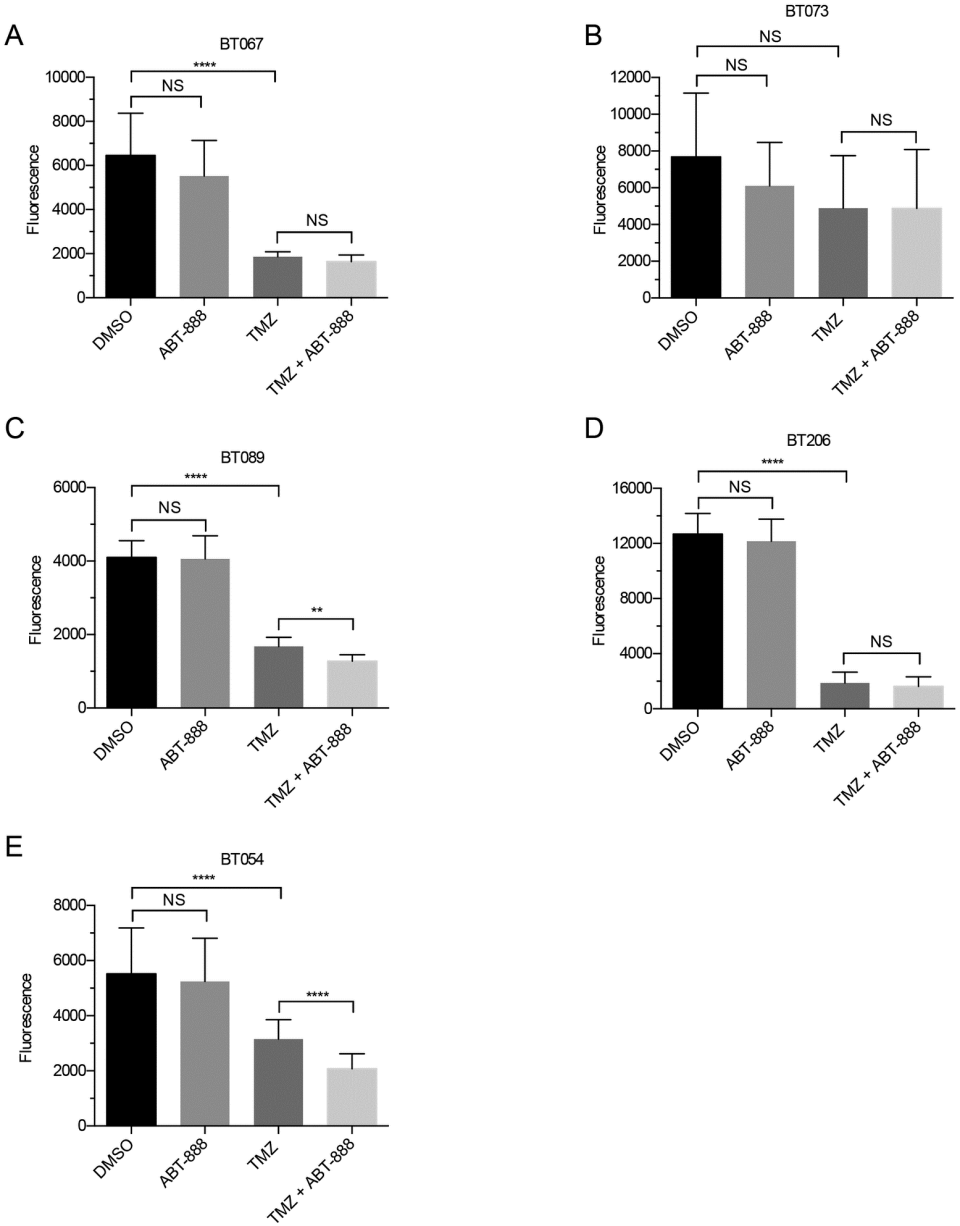
Average viability of Group I BTICs (n = 3, ± standard deviation or SD). Lines were treated with DMSO, ABT-888 (10 μM), TMZ (50 μM), or the combination of TMZ (50 μM) and ABT-888 (10 μM). Eight days after treatment cell viability was inferred using the alamarBlue^®^ assay. The unpaired t-test was applied to assess differences, as shown. Viability was unaffected by ABT-888 alone, whereas TMZ decreased viability in all lines but BT073 (B). The combination decreased viability in two of five BTICs (ns = p > 0.05; ** = p < 0.01; **** = p < 0.0001).

**Fig 2:**
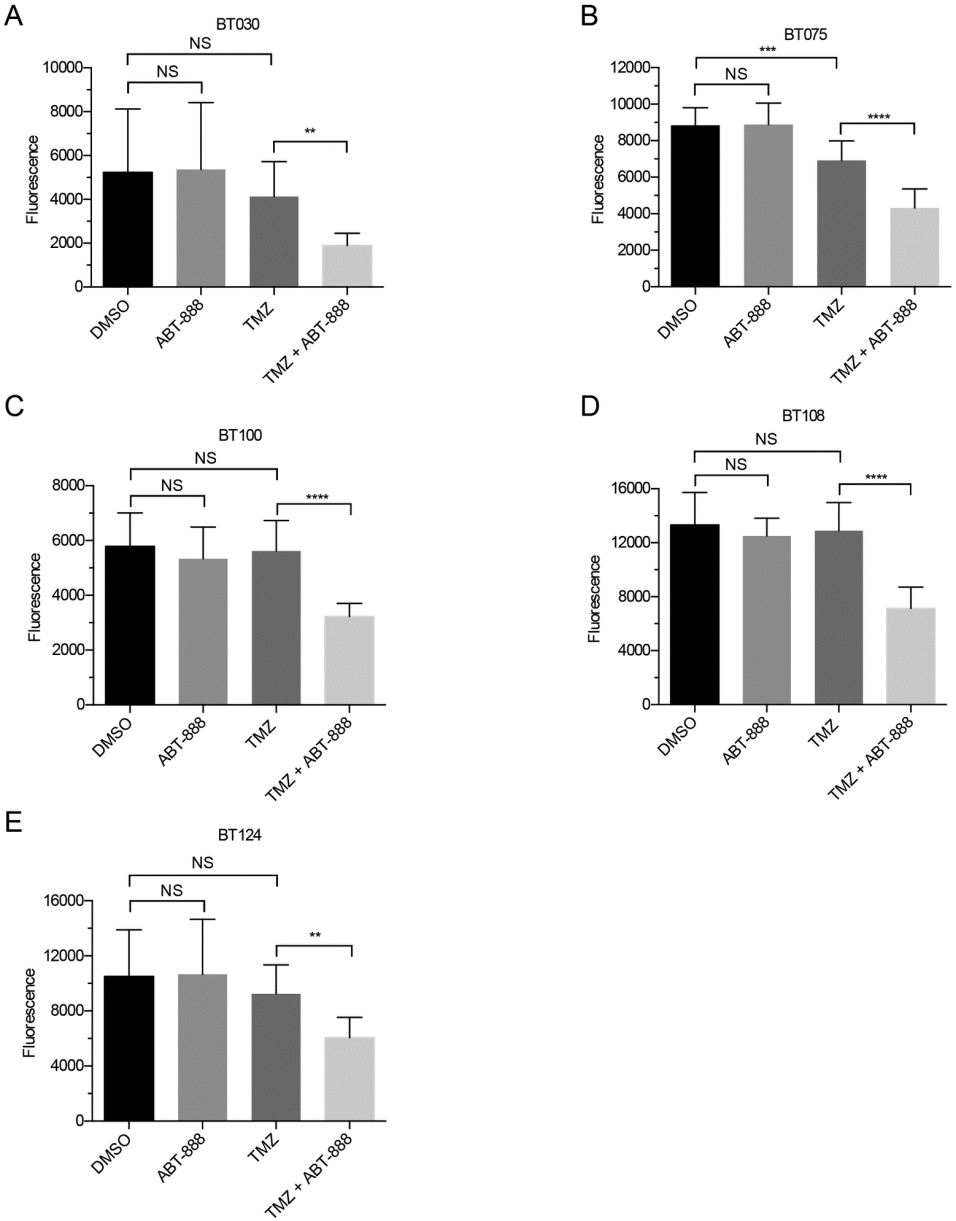
Average viability of Group II BTICs (n = 3, ± SD). Assessments and statistics were performed as in Fig 1. Viability was unaffected by ABT-888 or TMZ alone, except for BT075 (B). The combination of TMZ and ABT-888 significantly decreased viability in all BTICs (ns = p > 0.05; ** = p < 0.01; *** = p < 0.001; **** = p < 0.0001).

**Fig 3:**
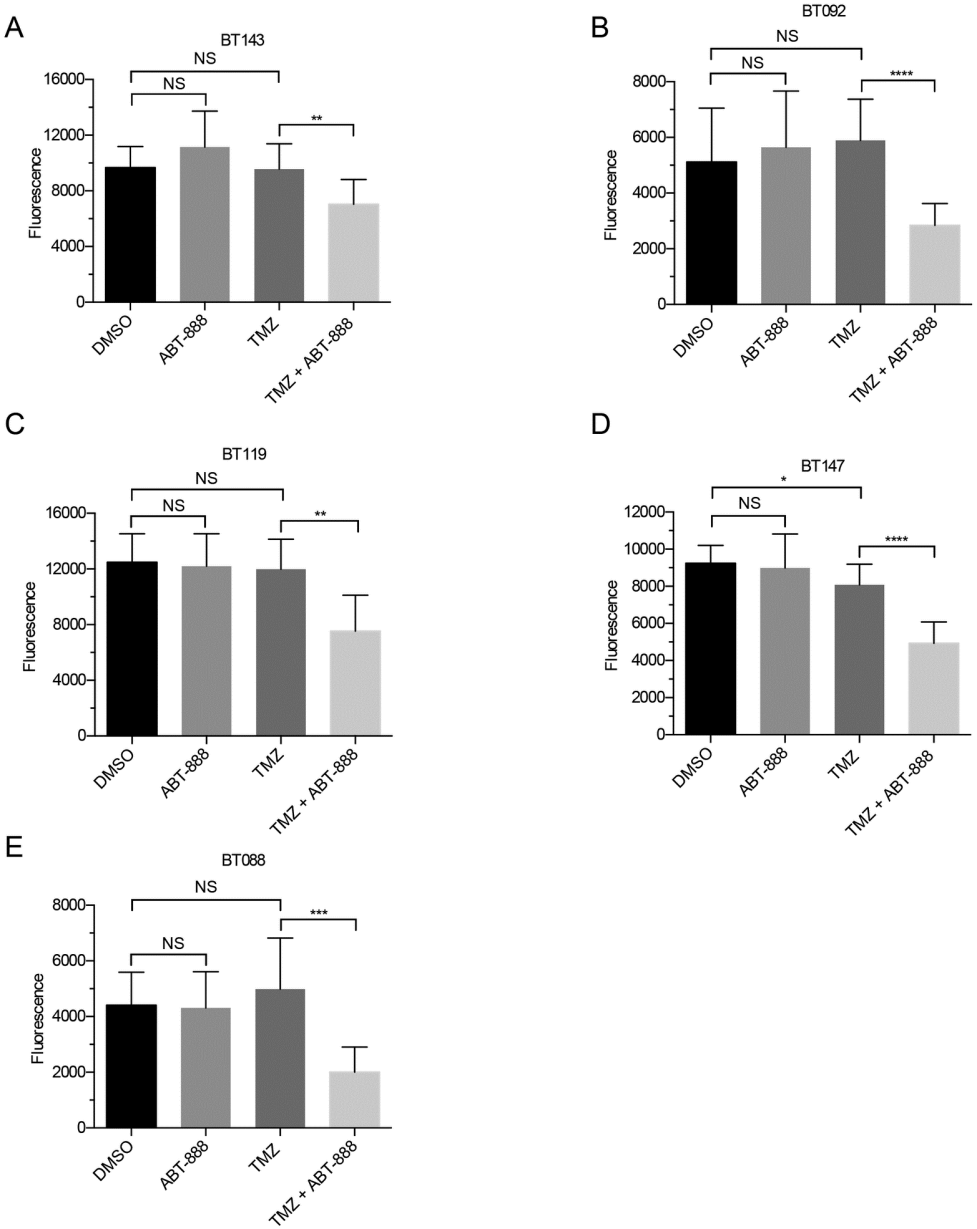
Average viability of Group III BTICs (n = 3, ± SD). Assessments and statistics were performed as in Fig 1. Viability was unaffected by ABT-888 alone and by TMZ alone, except for BT147 (D). The combination decreased viability in all BTIC lines (ns = p > 0.05; * = p < 0.05; ** = p < 0.01; *** = p < 0.001; **** = p < 0.0001).

**Fig 4:**
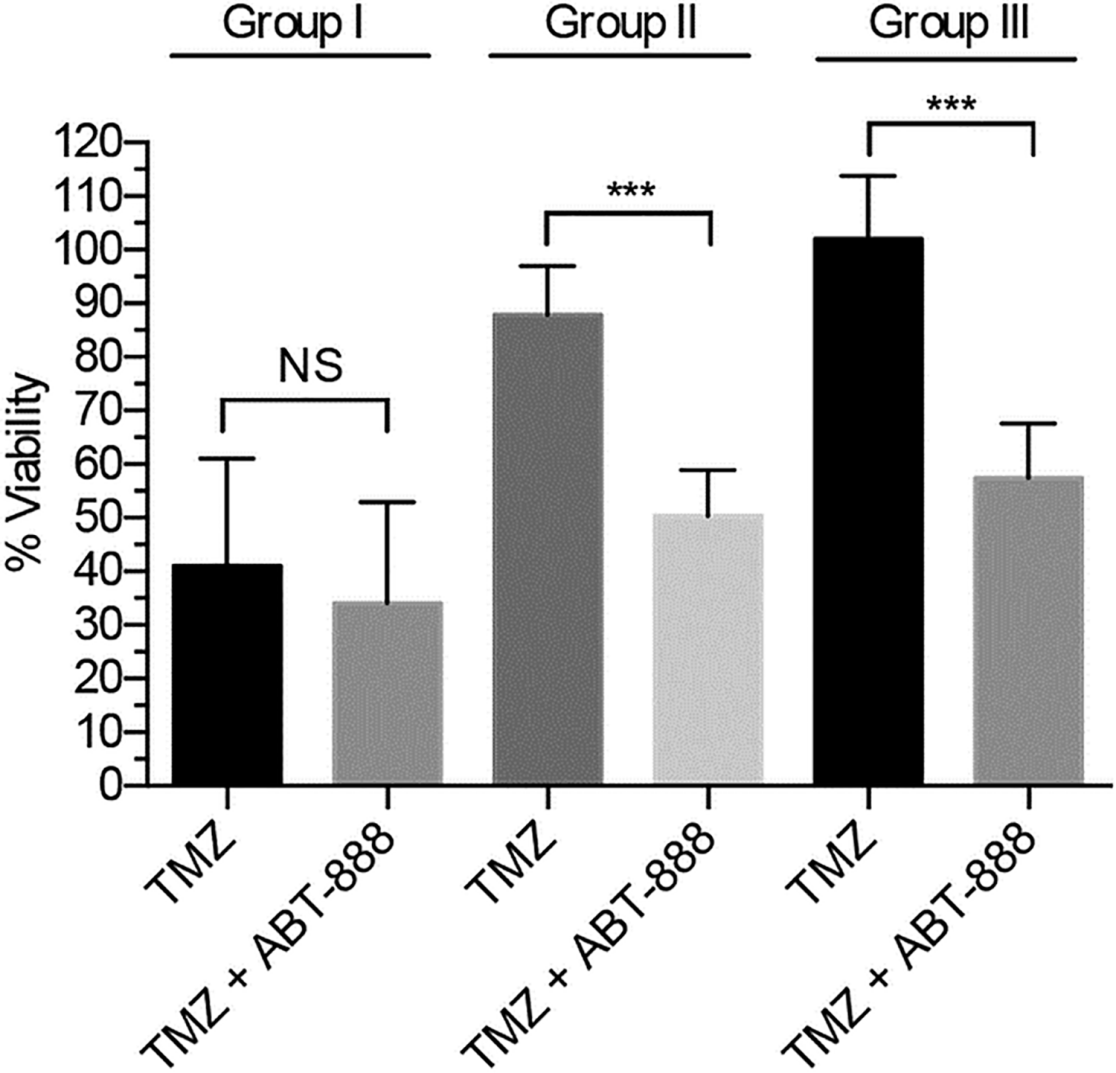
Average viability of BTICs in Group I *vs*. II *vs*. III (n = 3, ± SD). The unpaired t-test was applied to assess differences. On average, Group I was TMZ sensitive whereas Groups II and III were not. The addition of ABT-888 potentiated TMZ cytotoxicity in Groups II and III, but had no such potentiating effect in Group I (ns = p > 0.05; *** = p < 0.001).

TMZ (Sigma) and ABT-888 (Santa Cruz) were re-constituted in dimethylsulfoxide (DMSO; Sigma). For Figs 1–4 BTICs were plated at a density of 5000 cells/well in 96-well plates and 24 hours later treated with TMZ (50 μM), ABT-888 (10 μM), or both. For combined treatment, ABT-888 was added 2 hours before TMZ. Cell viability was measured after 8 days using the alamarBlue^®^ viability assay (Invitrogen). For S1 Fig, BTICs were plated and treated as described above and cell viability was measured over 14 days using the alamarBlue^®^ viability assay (Invitrogen).

### In vivo drug testing

Experiments were approved by the Animal Care Committee (#AC12-0066) at the University of Calgary and conducted as per standards established by the Canadian Council on Animal Care (‘Guide to the Care and Use of Experimental Animals’). BTICs were implanted into the striatum of SCID mice (*Mus musculus*; Charles River Laboratory) as previously described [27]. In brief, female, 6-8-week-old Fox Chase SCID mice over 17g (Charles River, C.B. - 17 SCID, strain 236) were anaesthetized with 175 mg/kg of Ketamine/Xylazine (19:1) via intraperitoneal injection. Additionally, 0.05 mg/kg of the analgesic Buprenorphine was given subcutaneously prior to surgery. Once in the anesthetic plane, mice were mounted on a stereotactic frame (Stoelting) and an 8 mm incision was made along the midline of the cranium to expose the skull. Using a micro-drill, a burr hole was made in the frontal bone, 1 mm anterior and 2 mm to the right of bregma. A 10 μL syringe (Hamilton) was then used to deliver 3 μL of cell suspension to a depth of 3 mm into the right striatum (stereotactic coordinates: 1.0 AP, 2.0 ML, 3.0 DV). The incision was closed with surgical staples and mice monitored while recovering on a heating pad until fully conscious. Following surgery, mice were offered recovery gel *ad libitum* and given a second dose of Buprenorphine 24 hours post-op. Fourteen days later mice were treated with TMZ (n = 8–10), ABT-888 (n = 8–10), or TMZ plus ABT-888 (n = 8–10). All drugs were suspended in Ora-Plus^®^ (Perrigo, USA). TMZ was administered by oral gavage at 30 mg/kg daily for 5 consecutive days (Monday through Friday) followed by a 2-day rest, and repeated weekly for 3 weeks. ABT-888 was given by oral gavage twice daily at 50 mg/kg per dose using the same schedule. For the combined treatment group, the first dose of ABT-888 was given concurrently with TMZ, and the second treatment 8 hours later. Control mice received Ora-Plus^®^ by oral gavage. Mice were monitored daily and euthanized by cervical dislocation when weight loss exceeded 20% of their initial weights, and/or when additional symptoms of illness were present, including lack of grooming, inactivity, and hunching. Brains were then removed and assessed for presence of a tumour via visual inspection as well as sectioning and staining with H&E to assess presence of tumour. All mice were euthanized after reaching the defined endpoints described above; there were no cases of sudden death.

### MSH6 shRNA knockdown

BT020, a BTIC line derived from a newly diagnosed methylated GBM, was grown adherently on poly-ornithine and laminin/entactin coated plates and transfected with shRNA lentivirus vectors designed to target *MSH6* using the ThermoFisher Scientific Expression Arrest protocol. Transfected cells were selected in 0.6 μg/ml puromycin for 10 days and maintained in 0.4 μg/ml puromycin. Knockdown was confirmed by Western blotting (procedure described below; S3A Fig). To validate the sensitivity of the BT020shNeg (MSH6 wild-type) and the BT020shMSH6 (MSH6 knockdown) lines to TMZ (S3B Fig), cells were treated with increasing concentrations of TMZ and viability was measured after 7 days using the WST-1 assay (Sigma Aldrich). To assess the response of BT020shMSH6 cells to the combination of TMZ and ABT-888 (S3C Fig), cells were treated with one dose of DMSO, TMZ (100 μM), ABT-888 (100 μM) or the combination of TMZ (100 μM) and ABT-888 (100 μM), and after 5 days viability was measured using the WST-1 assay (Sigma Aldrich). [A higher concentration of TMZ was used in the mechanistic studies to ensure the detection of DNA strand breaks in the Comet assay.]

### Comet assay

BT020shNeg and BT020shMSH6 cells were treated with DMSO, TMZ (100 μM), ABT-888 (100 μM), or the combination of TMZ (100 μM) and ABT-888 (100 μM) for 4 hours, re-suspended in agarose at 1x10^5^ cells/mL and placed on CometSlides in duplicates using the CometAssay kit (Trevigen). Slides were denatured in alkali solution, electrophoresed, and stained with SYBR Green (1:10000). Randomly selected comets (n = 50) were scored with image-analysis software Comet IV (Perceptive Instruments Ltd.).

### Western blot

Cells were treated with DMSO, TMZ (100 μM), ABT-888 (100 μM), or the combination of TMZ (100 μM) and ABT-888 (100 μM) and harvested at various time points. Whole cells were lysed in standard NP-40 lysis buffer (Life Technologies) with 1X protease inhibitors tablet, 0.1 mM NaVO_3_, 1 mM DTT, and 1 μM PMSF (Roche Applied Science). Samples were subjected to SDS-PAGE gel electrophoresis using standard protocols transferring 30 μg of protein onto a PVDF membrane (Millipore), followed by blocking with 10% nonfat milk powder for 30 min and overnight incubation at 4°C with either anti-MSH6 (Cat# 610918, BD Biosciences), anti-actin (Cat# MAB1501R Millipore), phospho-CHK1 (Ser 317, Cat# 12302S, Cell Signaling Technologies), CHK1 (Cat# 2360S, Cell Signaling Technologies) phospho-histone H2A.X (Ser 139, 80312S, Cell Signaling Technologies), or H2A.X (Cat# 2595S, Cell Signaling Technology) occording to manufacturer’s specifications. Membranes were incubated with appropriate secondary antibody for 1 h followed by enhanced chemiluminescence visualization and substrate detection (Thermo Scientific).

### DNA replication

Cells were grown and treated as described under “Comet Assay” and “Western Blot”. Staining was performed by incubating in 10 μM EdU for 2 hours after which cells were washed, fixed, and stained with Hoescht per manufacturer’s protocol (Invitrogen). Cells were analyzed using a BD LSRII flow cytometer.

### Statistical analyses

Graphpad Prism was used for all analyses. The unpaired t-test was conducted to compare BTIC viability between treatment groups. The log-rank (Mantel-Cox) test was used to compare Kaplan-Meier survival curves under different treatment conditions.

### Sequencing

Genome sequencing was used to identify gene mutations in Table 1 in BTICs, and performed at the Genome Sciences Centre of the British Columbia Cancer Agency (Vancouver, BC).

**Table 1:** Description and clinical subgroups of the patient-derived BTICs.

Clinical Group	BTIC Identifier	Pathologic Diagnosis	Gender	Age at Diagnosis	Prior Treatment	*MGMT* Status	*MSH6* Status	*IDH1 Status*	*EGFR Status*	*PTEN* Status	*p53* Status
**I**	BT067	GBM	M	44	None	M	WT	WT	WT	HET	WT
**Newly diagnosed, MGMT-methylated**	BT073	GBM	M	52	None	M	NT	WT	VIII	MT	MT
	BT089	GBM	F	60	None	M	WT	WT	WT	MT	WT
	BT206	GBM	M	68	None	M	NT	WT	WT	WT	MT
	BT054	Oligo	F	49	None	M	WT	MT	WT	WT	WT
**II**	BT030	GBM	M	67	None	UM	NT	WT	WT	MT	WT
**Newly diagnosed, MGMT-unmethylated**	BT075	GBM	M	74	None	UM	WT	WT	WT	WT	WT
	BT100	GBM	M	63	None	UM	WT	WT	WT	MT	WT
	BT108	GBM	M	46	None	UM	NT	WT	MT	WT	WT
	BT124	GBM	NA	NA	None	UM	NT	WT	WT	MT	MT
**III**	BT143	GBM	F	39	TMZ/RT	M	MT	WT	WT	WT	WT
**Recurrent**	BT092	GBM	M	23	TMZ/RT	UM	MT	WT	WT	MT	MT
	BT119	GBM	F	69	TMZ/RT	UM	MT	WT	MT	WT	MT
	BT147	GBM	M	55	TMZ/RT	UM	MT	WT	VIII	MT	MT
	BT088	Oligo	M	33	TMZ/RT	M	MT	WT	WT	WT	MT

Abbreviations: GBM = Glioblastoma; Oligo = Oligodendroglioma; None = no prior therapy; TMZ/RT = temozolomide and radiotherapy; M = MGMT promoter methylated; UM = MGMT; unmethylated; WT = wildtype; MT = mutant; NT = not tested; VIII = VIII mutant; NA = not available

## Results

### ABT-888 reverses TMZ-resistance in BTICs

To determine if PARP inhibition sensitized gliomas to TMZ, we treated 15 low-passage patient-derived lines established and grown as spheres in neural stem cell media [27] (Table 1) with TMZ, ABT-888, or the combination. Group I lines were derived from newly diagnosed, treatment-naïve, *MGMT* methylated, high-grade gliomas (GBM and oligodendroglioma); Group II lines were derived from newly diagnosed, treatment-naïve, unmethylated high-grade gliomas (GBM only); and Group III lines were derived from high-grade gliomas that had recurred after treatment with TMZ and RT (GBM and oligodendroglioma). *MSH6* mutation status (wild type or mutant) was also assessed because mutations are frequently seen in high-grade gliomas that recur after treatment with TMZ and RT, and because *MSH6* mutations are known to mediate TMZ resistance [30–32]. After showing that single agent ABT-888 did not alter the viability of any BTIC in any Group, we proceeded to test the responses of different BTICs (Groups I, II, III) to TMZ alone and to TMZ plus ABT-888.

Newly diagnosed methylated BTICs in Group I were sensitive to TMZ with responses that ranged from a 62% decrease (p < 0.0001) in the viability of the oligodendroglioma line BT054 to an 87% decrease (p < 0.0001) in the viability of the GBM line BT206 (Fig 1). In these intrinsically sensitive methylated lines, the addition of ABT-888 to TMZ had a minor incremental effect on TMZ sensitivity in two of five lines only; the viability of BT089 decreased by an additional 10% (p = 0.010) and the viability of BT054 decreased by a further 20% (p < 0.0001; Fig 1). These changes were minimal compared to TMZ alone.

BTICs in Groups II and III responded differently than Group I. As expected, based on MGMT status, Group II lines were largely resistant to TMZ; only one line, BT075, displayed a significant decrease in viability after treatment (p = 0.001; Fig 2). Also in contrast to Group I, all Group II lines were significantly more sensitive to TMZ after co-treatment with ABT-888. The degree of sensitization ranged from 30% in BT075 (p < 0.0001) to 43% in BT108 (p < 0.0001; Fig 2). Group III lines with *MSH6* mutations were resistant to TMZ, the sole exception being BT147, which displayed a 13% decrease (p = 0.0304) in viability after exposure to TMZ (Fig 3). Like group II, Group III lines were sensitized to TMZ by co-treatment with ABT-888; decreases in viability ranged from 34% in BT147 (p < 0.0001) to 67% in BT088 (p = 0.0005; Fig 3).

The data for each group were then averaged to reveal trends in response across clinically distinct sets of BTICs (Fig 4). In methylated Group I lines, the average decrease in viability after TMZ was 59% (p = 0.0018), which decreased by only 7% (NS) following the addition of ABT-888. In unmethylated Group II lines, the average decrease in viability after TMZ was 12% (NS) compared to 50% (p < 0.001) after the addition of ABT-888. In Group III lines that had acquired resistance, viability increased by 2.1% (NS) after exposure to TMZ but then decreased by 42% (p < 0.001) following the addition of ABT-888. These data show that PARP inhibition overcomes resistance to TMZ in patient derived BTICs that are intrinsically resistant to TMZ (Group II) or have acquired resistance (Group III), but has less effect on lines that are intrinsically sensitive to TMZ (Group I).

To assess whether these effects were sustained, we monitored the 14-day viability of representative BTICs from each group, and saw the same patterns. BT089, a Group I line, was sensitive to TMZ (p < 0.0001), whereas BT100 from Group II and BT088 from Group III were resistant (p = 0.67 and p = 0.13, respectively). The addition of ABT-888 did not increase TMZ sensitivity in BT089 (p = 0.72), but restored sensitivity in BT100, BT092 and BT088 (p < 0.001; p < 0.0001; and p < 0.0001, respectively). These data show that *in vitro* responses to a single dose of TMZ and a single dose of TMZ and ABT-888 persisted over time (S1 Fig).

### Co-treatment prolongs the survival of mice bearing TMZ resistant xenografts

We then tested whether ABT-888 potentiated TMZ *in vivo*, and whether the patterns of response were similar to those observed *in vitro*. To maximize relevance, tumor-bearing mice were treated with concentrations and schedules of TMZ that mirrored those used clinically [16, 33, 34]. To assess tolerability, we tracked the weights of mice implanted with a representative cell line and observed that mice treated with TMZ or TMZ plus ABT-888 maintained stable weight throughout the three week treatment period (S2 Fig), demonstrating treatments were well tolerated.

**Fig 5:**
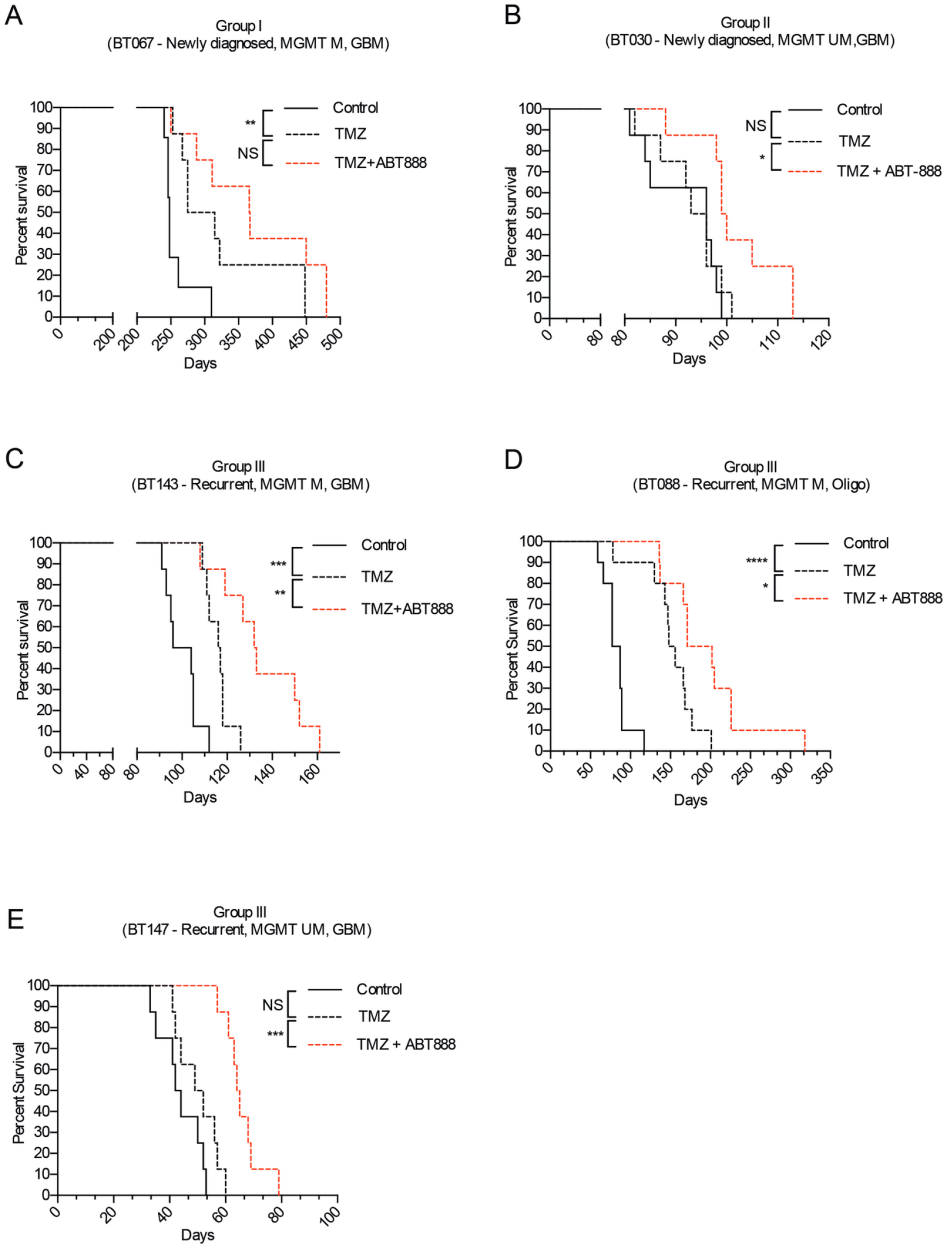
Kaplan-Meier survival curves of mice treated with DMSO, TMZ, or TMZ and ABT-888. Mice were implanted with BTICs from Groups I, II and III. Mice bearing BT067 (A), a Group I line, lived significantly longer after TMZ, but had no further survival benefit from the addition of ABT-888 to TMZ. Mice bearing BT030 (B), a Group II line, did not live longer after TMZ alone, but lived significantly longer after the addition of ABT-888 to TMZ. Mice bearing BT143 and BT088 (C, D), methylated lines from Group III, lived significantly longer after TMZ and longer still after the addition of ABT-888 to TMZ. Mice bearing the BT147 (E), a Group III unmethylated line, did not live significantly longer after TMZ, but had significantly longer survival after the addition of ABT-888 to TMZ. The log-rank (Mantel-Cox) test was used to compare survival curves (ns = p > 0.05; * = p < 0.05; ** = p < 0.01; *** = p < 0.001; **** = p < 0.0001).

Next, we measured the survival of tumor-bearing mice treated with TMZ alone. As seen *in vitro*, the survival of mice engrafted with lines from Groups I and II was MGMT-dependent; mice bearing BT067, a methylated line, lived longer after treatment with TMZ than control-treated mice (295 *vs*. 248 days, p = 0.004; Fig 5A), while those bearing BT030, an unmethylated line, derived no survival benefit from treatment with TMZ (94.5 *vs*. 96 days, p = 0.7; Fig 5B). In contrast, the *in vitro* and *in vivo* responses to TMZ among Group III lines were sometimes discordant, and despite having mutations in *MSH6*, lines from Group III displayed degrees of MGMT dependence. Mice engrafted with the recurrent methylated GBM, BT143, lived longer after TMZ (116.5 *vs*. 100 days, p = 0.0006; Fig 5C) as did mice bearing BT088, the methylated oligodendroglioma line (152 *vs*. 82 days, p < 0.0001; Fig 5D), whereas those implanted with the recurrent unmethylated GBM line, BT147, derived no benefit from treatment with TMZ (50.5 *vs*. 43 days, p = 0.09; Fig 5E).

Adding ABT-888 to TMZ also reversed resistance to TMZ *in vivo* (Fig 5 and S1 Table). Significant increases in survival were only seen in mice bearing xenografts from newly diagnosed unmethylated GBMs (Group II) and *MSH6* mutated recurrent tumors (Group III). Although there was a trend to longer survival in mice bearing xenografts from newly diagnosed methylated tumors (Group I), the observed differences did not reach statistical significance, again suggesting that PARP inhibition may have a less profound effect on TMZ-sensitive tumors. ABT-888 alone had no effect on the survival of tumor-bearing mice and did not cause serious toxicity.

### ABT-888 restores TMZ-induced DNA strand breakage and re-engages the G2/M checkpoint

To explore the mechanisms by which AT-888 potentiates TMZ in the context of *MSH6* inactivation, we used lentiviral shRNA to knock down *MSH6* in BT020, a line derived from a newly diagnosed, *MGMT* methylated GBM patient. BT020shMSH6 (MSH6-kd) had undetectable levels of MSH6 compared to BT020shNeg (MSH6-wt; S3A Fig). Consistent with *MSH6* mutant lines, MSH6-kd cells demonstrated robust TMZ resistance across a range of doses (S3B Fig) and could be re-sensitized to TMZ by co-treatment with ABT-888 (S3C Fig). ABT-888 as a single agent had no discernible effect on the viability of MSH6-kd cells.

**Fig 6:**
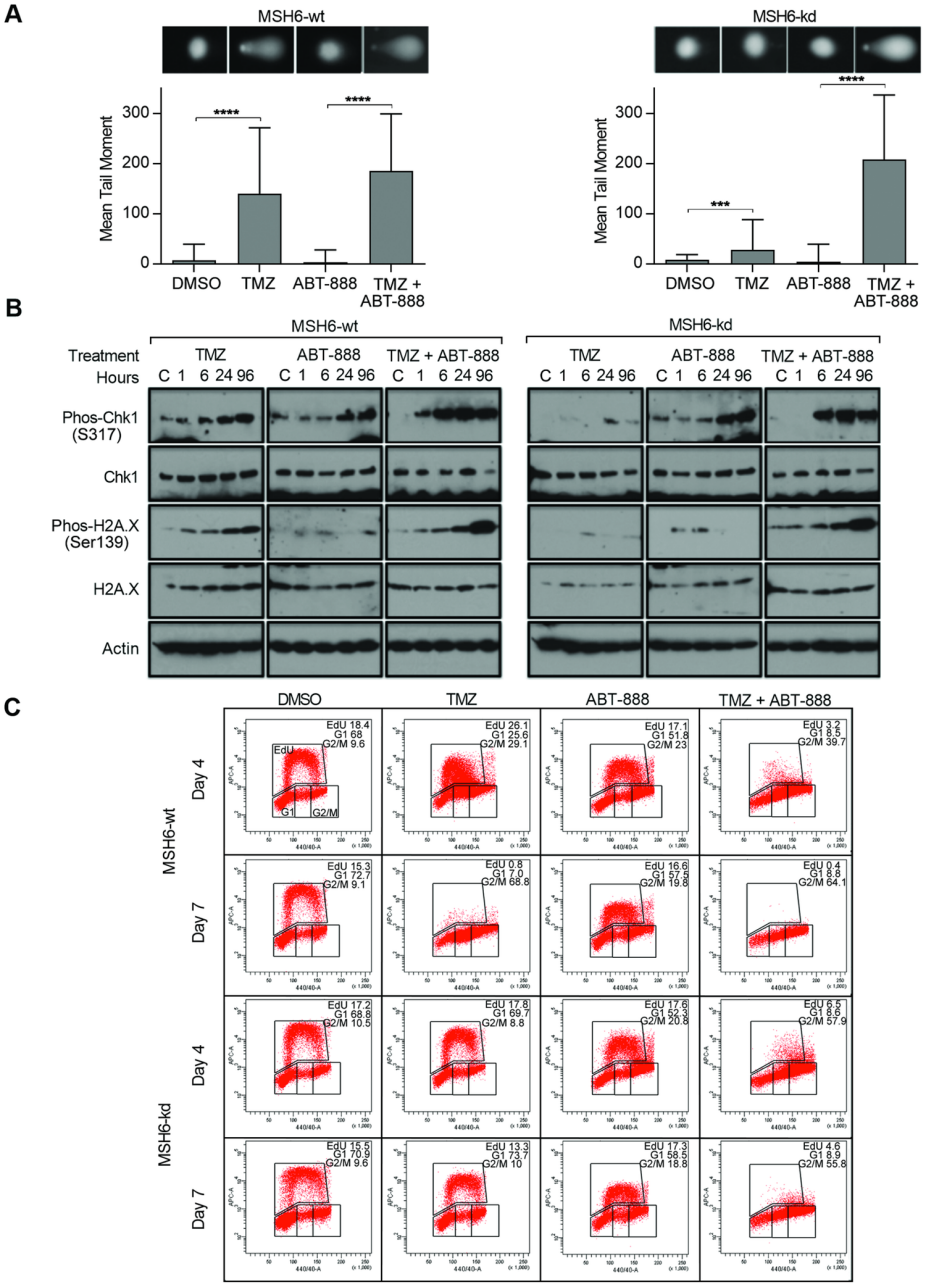
TMZ plus ABT-888 restores DNA breaks, G2/M checkpoint activation, and synthesis arrest in *MSH6* mutant lines. **(A)** Alkaline comet assay representing the mean tail moments of 50 samples grown in suspension and treated with DMSO, TMZ (100 μM), ABT-888 (100 μM), or TMZ (100 μM) and ABT-888 (100 μM). Samples were stained with SYBR green and analyzed by epiflourescence microscopy; representative images are displayed. (B) Lysates taken from MSH6-wt and MSH6-kd cells were treated with DMSO, TMZ (100 μM), ABT-888 (100 μM), or TMZ (100 μM) and ABT-888 (100 μM) and a control sample collected immediately previous to treatment (Control = C), or 1h, 6h, 1d, and 3d after drug exposure. Western blotting was performed for total and phos-CHK1, total and phos-H2A.X, and actin (controls). (C) MSH6-wt and MSH6-kd cells were grown in suspension and exposed to DMSO, TMZ (100 μM), ABT-888 (100 μM), or TMZ (100 μM) and ABT-888 (100 μM). At days 4 and 7 after treatment cells were stained with EdU and analyzed on the BD LSRII flow cytometer.

Aware that the cytotoxic effects of TMZ are mediated through DNA strand breakage we used the alkaline comet assay to assess DNA damage in MSH6-wt and MSH6-kd cells under different treatment conditions. As measured by the mean tail moment, we observed that DNA damage persisted when MSH6-wt cells were exposed to TMZ alone, but scarcely detectable in MSH6-kd cells, confirming that MMR activity is necessary for the induction of strand breaks (Fig 6A). Following co-treatment with TMZ and ABT-888, there was a slight increase in DNA damage in MSH6-wt cells as compared to the TMZ alone condition. In contrast, there was a substantial increase in DNA damage in MSH6-kd cells. These findings are consistent with the interpretation that PARP inhibition increases DNA damage in the setting of MMR inactivation. ABT-888 as a single agent had no effect on the extent of DNA damage in either MSH6-wt or MSH6-kd cells during the 3 days of drug treatment.

The DNA damage response is known to involve signaling through phosphorylated CHK1 and γH2AX, and to be associated with the formation of DNA breaks [35, 36]. In our isogenic model, exposure to TMZ was accompanied by a rapid accumulation of phosphorylated CHK1 and γH2AX in MSH6-wt cells, whereas activation of the DNA damage response by this measure was not seen in MSH6-kd cells (Fig 6B). However, co-treatment with ABT-888 re-engaged the checkpoint signaling response in MSH6-kd cells, as revealed by an increase in phosphorylated CHK1 and γH2AX. Thus, ABT-888-mediated enhancement of TMZ cytotoxicity in an MMR-deficient background is associated with re-engagement of a common pathway of DNA damage checkpoint signaling. Exposure to ABT-888 alone was followed by a minor increase in phosphorylated CHK1, perhaps related to progression of cells through the cell cycle and accumulation in G2/M [37] and had no effect on the phosphorylation of γH2AX.

We then used Edu incorporation to assess the outcome of TMZ-induced damage and checkpoint signaling on DNA replication (Fig 6C). In MSH6-wt cells, exposure to TMZ led to a reduction in replication by day 4 and complete absence by day 7, in an addition to a G2/M arrest. In stark contrast, in MSH6-kd cells TMZ exposure had no effect on DNA replication on day 4 or 7. While ABT-888 alone did not alter Edu incorporation in either MSH6-wt or MSH6-kd cells, addition of ABT-888 to TMZ restored the halt in DNA replication and G2/M arrest in MSH6-kd cells.

## Discussion

The emergence of TMZ as a life prolonging therapy for patients with newly diagnosed GBM is a major milestone in brain tumor care, but unfortunately, 50% of newly diagnosed patients derive little benefit, and those that respond, relapse with resistant tumors. Similar concerns apply to oligodendroglioma where most newly diagnosed tumors respond to lomustine (with procarbazine and vincristine; PCV), but relapsing cases have acquired resistance. Resistance to TMZ and PCV is problematic because there are no highly effective second-line therapies for either GBM or oligodendroglioma [38, 39]. To improve the efficacy of chemotherapy for these patients, we asked whether PARP inhibition might be helpful. To mimic clinical situations encountered by oncologists, we studied the viability of cells and xenografts from newly diagnosed methylated tumors, newly diagnosed unmethylated tumors, and recurrent tumors with *MSH6* mutations.

We began by assessing whether sensitivity to TMZ in these models would recapitulate the patient experience, where sensitivity and resistance are MGMT dependent [28]. We observed that BTICs and xenografts from newly diagnosed tumors responded as expected; those that were methylated were sensitive to TMZ, and those that were unmethylated relatively resistant. The effect of MGMT on response was variable in models of recurrent tumors; BTICs were resistant irrespective of their methylation status, but the effect from *MGMT* methylation was still evident in orthotopic models. Xenografts derived from unmethylated recurrent cases were resistant to TMZ, whereas those from methylated recurrences retained a degree of sensitivity to TMZ. These results are generally consistent with clinical experience, wherein most patients with recurrent high-grade gliomas are resistant to TMZ, but some with recurrent methylated tumors respond a second time, albeit briefly. These results suggest that BTIC and orthotopic xenograft models of GBM and oligodendroglioma can be used to study potential roles for PARP inhibition in the treatment of high-grade glioma.

Using this approach, we found that BTICs and xenografts that were intrinsically resistant or became resistant to TMZ through the acquisition of *MSH6* mutations were sensitized by co-treatment with ABT-888, but surprisingly, adding ABT-888 to TMZ did not magnify the intrinsic sensitivity of models of newly diagnosed methylated high-grade gliomas. Additionally, we demonstrate that restoring sensitivity to TMZ with ABT-888 was associated with re-appearance of DNA strand breaks, re-engagement of the G2/M checkpoint, and interruption of DNA synthesis. Taken together, these data suggest that PARP inhibition may be especially helpful in situations where gliomas are or become resistant to TMZ.

How do we explain this result? Why are resistant cells preferentially sensitized? In resistant cells, the toxic adducts on O6-guanine are either repaired by MGMT or tolerated in *MSH6* mutant cells, and consequently, the cytotoxic effects of the TMZ-induced N3 and N7 methyl adducts govern cell fate. Unlike O6-guanine lesions that are repaired by MGMT, those at N3-adenine and N7-guanine are reversed by BER, a PARP dependent system. Thus, in resistant cells, PARP inhibition leads to the accumulation of N3 and N7 adducts, which trigger DNA strand breaks and apoptosis [40, 41]. In sensitive cells, the overwhelming cytotoxicity of O6 lesions may obscure any contribution to cell death by secondary adducts, explaining why there appears to be less incremental tumor cell killing when ABT-888 is combined with TMZ in newly diagnosed methylated cases [3, 4].

Might our findings point to treatment strategies for gliomas that incorporate PARP inhibition, as is now standard for patients with breast and ovarian cancers that are associated with mutations of *BRCA1* and *BRCA2*? Unfortunately, recent data from early phase clinical trials in children and adults with recurrent gliomas suggest that the combination of TMZ and ABT-888 may not have the same efficacy in patients with gliomas. Su *et al* [34] saw no complete or partial responses to ABT-888 and TMZ in a phase I trial of new and recurrent high-grade gliomas in children. Likewise, Robins *et al* [42] reported a mere 3% complete and partial response rate in a phase I/II study of recurrent tumors in adults. Notwithstanding long periods of stable disease in some patients [34], these results are discouraging. Moreover, many of the patients in these trials had previously been exposed to TMZ, the setting in which our data with cells and xenografts would have predicted benefit from ABT-888.

Can we reconcile these negative trials with our data suggesting that co-treatment with ABT-888 kills cells from GBMs and oligodendrogliomas that are resistant to TMZ? What clinical situations might be better managed by a drug combination with these features? Our data point to patients with recurrent tumors as one subset that might benefit, but recurrent GBMs are aggressive and re-treatment may not alter their poor prognosis. Perhaps, as our data suggest, co-treatment with a PARP inhibitor would reverse intrinsic resistance in newly diagnosed cases that express MGMT; such patients currently receive TMZ, but are not especially well served by this standard of care. Although our data do not identify patients with newly diagnosed methylated GBMs, already sensitive to TMZ, as a subgroup likely to be become ‘supersensitive’ with the addition of a PARP inhibitor to TMZ, the possibility that they will also benefit is being tested in a trial that assesses progression-free survival after TMZ and ABT-888 in methylated GBMs (NCT02152982).

Perhaps there are other situations in which the care of patients with gliomas can be improved by adding a PARP inhibitor. Recent success in treating BRCA1/2 deficient cancers, and our data showing that resistant cells with MMR mutations are sensitive to TMZ and ABT-888, leads us to speculate that treatment of sensitive tumours with a PARP inhibitor might thwart the emergence of MMR-mediated TMZ resistance. For example, resistance undermines the treatment of patients with low-grade gliomas in whom TMZ might be used to defer RT. In this scenario, co-treatment with a PARP inhibitor potentially creates a synthetic lethal interaction in which resistant cells are eliminated preemptively as they appear in the tumor. In this way, resistance to TMZ is suppressed, tumor control is improved, and the lifespan of a well-tolerated and useful chemotherapy is extended.

## Supporting information

S1 Fig Average viabilities of BTIC lines over 14 days after a single exposure to TMZ (50 μM), ABT-888 (10 μM), or the combination of TMZ and ABT-888. Results are normalized to DMSO treated BTICs for BT089 from Group I (A); BT100 from Group II (B); and BT092 and BT088 from Group III (C, D). Viability was inferred using the alamarBlue^®^ assay. Responses were sustained over 14 days and followed the patterns characteristic of each group as seen in Fig 4 (ns = p > 0.05; *** = p < 0.001; **** = p < 0.0001).
(TIF)

S2 Fig Weights of mice engrafted with a representative line monitored during treatment. Mice were treated with (A) DMSO, (B) TMZ (30 mg/kg once daily), or (C) TMZ (30 mg/kg once daily) and ABT-888 (50 mg/kg twice daily) for 5 consecutive days, followed by a 2 day rest, and repeated weekly for 3 weeks. Mice were weighed every 3 days for 60 days. Treatments were well tolerated.
(TIF)

S3 Fig An MSH6-inactivated BTIC line demonstrates TMZ resistance and restoration of sensitivity when co-treated with ABT-888. A MSH6 null line was developed by transfecting BT020 with a non-targeting control vector or lentivirus shRNA against *MSH6*. The resulting lines BT020shNeg (MSH6-wt) and BT020shMSH6 (MSH6-kd) were then checked for MSH6 expression using Western blotting (A), and demonstrated absence of MSH6 expression in the knockdown line. (B) BT020, MSH6-wt, and MSH6-kd were cultured with increasing concentrations of TMZ and viability measured after 7 days using the WST-1 assay. BT020 and MSH6-wt were sensitive to TMZ, whereas MSH6-kd was resistant. (C) MSH6-kd were treated with DMSO, TMZ (100 μM), ABT-888 (100 μM), or TMZ+ABT-888 and viability measured after 5 days using the WST-1 assay. Addition of ABT-888 to TMZ restored sensitivity in the MSH6-kd line.
(TIF)

S1 Table Survival data for tumor bearing mice that were treated as described. (TIF)
