# Supplementary figures and images for: ABT-888 restores sensitivity in temozolomide resistant glioma cells and xenografts
Source: PLoS One. 2018 Aug 28;13(8):e0202860. doi: 10.1371/journal.pone.0202860 (PMC6112648; doi:10.1371/journal.pone.0202860)

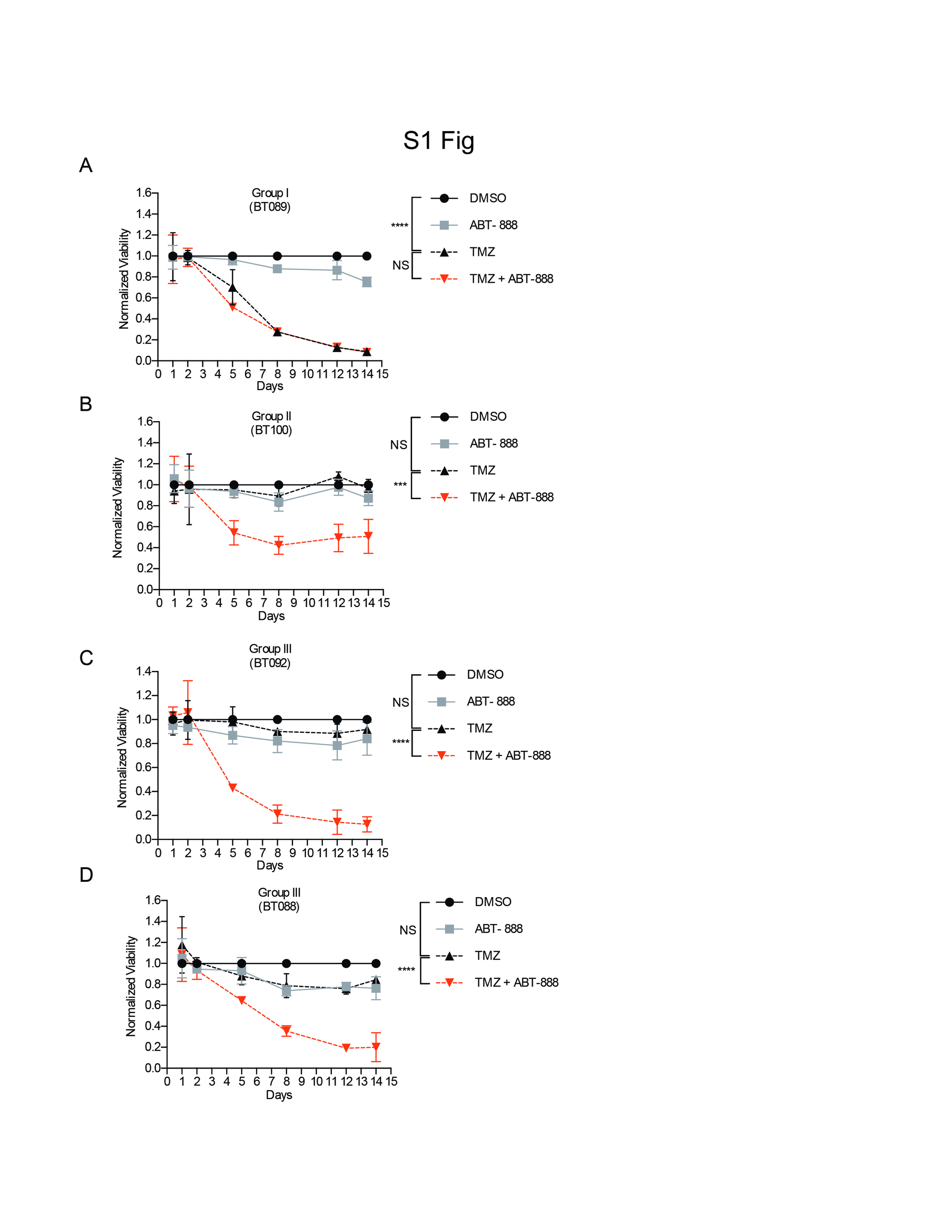

Supplement: S1 Fig — Results are normalized to DMSO treated BTICs for BT089 from Group I (A); BT100 from Group II (B); and BT092 and BT088 from Group III (C, D). Viability was inferred using the alamarBlue® assay. Responses were sustained over 14 days and followed the patterns characteristic of each group as seen in Fig 4 (ns = p > 0.05; *** = p < 0.001; **** = p < 0.0001). (TIF) [file pone.0202860.s001.tif]

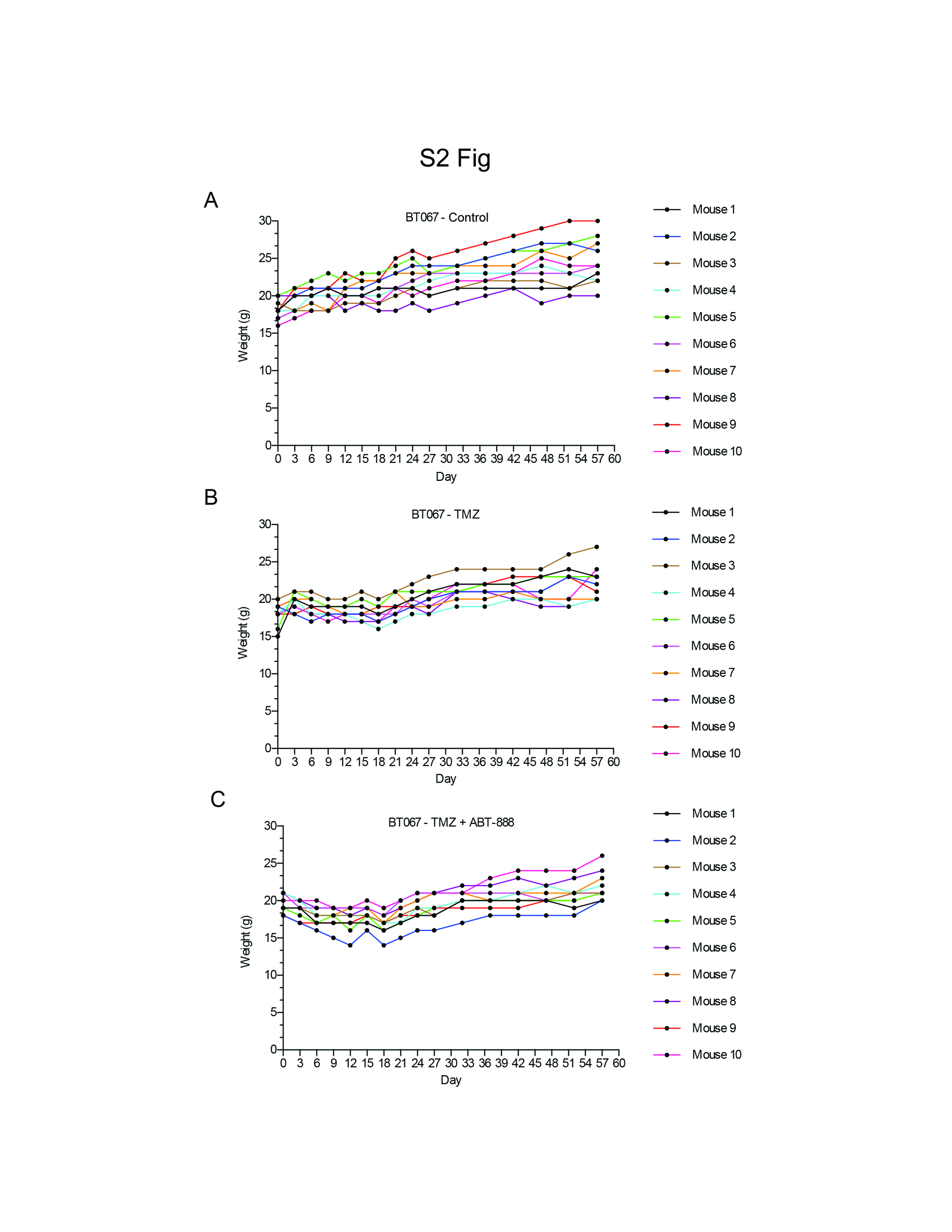

Supplement: S2 Fig — Mice were treated with (A) DMSO, (B) TMZ (30 mg/kg once daily), or (C) TMZ (30 mg/kg once daily) and ABT-888 (50 mg/kg twice daily) for 5 consecutive days, followed by a 2 day rest, and repeated weekly for 3 weeks. Mice were weighed every 3 days for 60 days. Treatments were well tolerated. (TIF) [file pone.0202860.s002.tif]

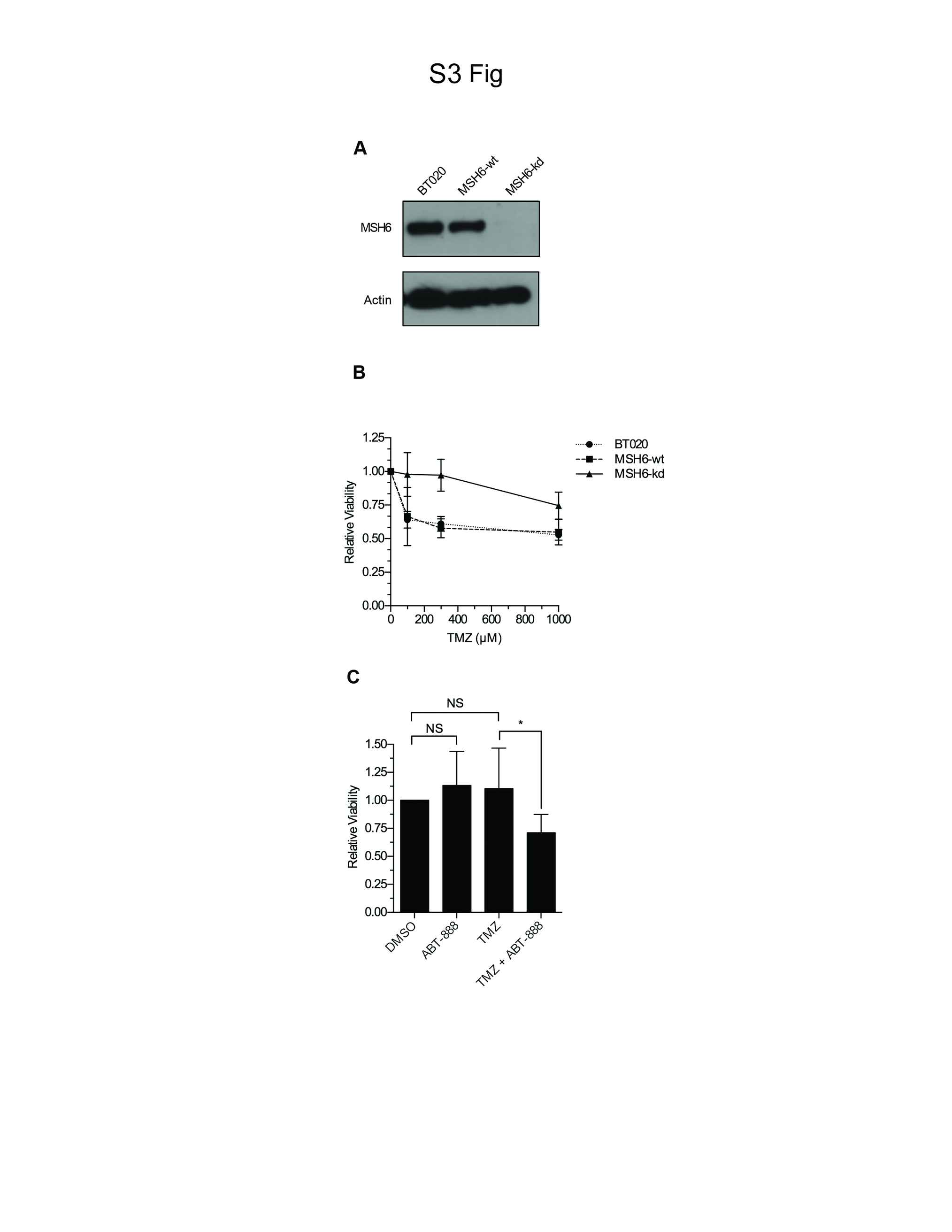

Supplement: S3 Fig — A MSH6 null line was developed by transfecting BT020 with a non-targeting control vector or lentivirus shRNA against MSH6. The resulting lines BT020shNeg (MSH6-wt) and BT020shMSH6 (MSH6-kd) were then checked for MSH6 expression using Western blotting (A), and demonstrated absence of MSH6 expression in the knockdown line. (B) BT020, MSH6-wt, and MSH6-kd were cultured with increasing concentrations of TMZ and viability measured after 7 days using the WST-1 assay. BT020 and MSH6-wt were sensitive to TMZ, whereas MSH6-kd was resistant. (C) MSH6-kd were treated with DMSO, TMZ (100 μM), ABT-888 (100 μM), or TMZ+ABT-888 and viability measured after 5 days using the WST-1 assay. Addition of ABT-888 to TMZ restored sensitivity in the MSH6-kd line. (TIF) [file pone.0202860.s003.tif]

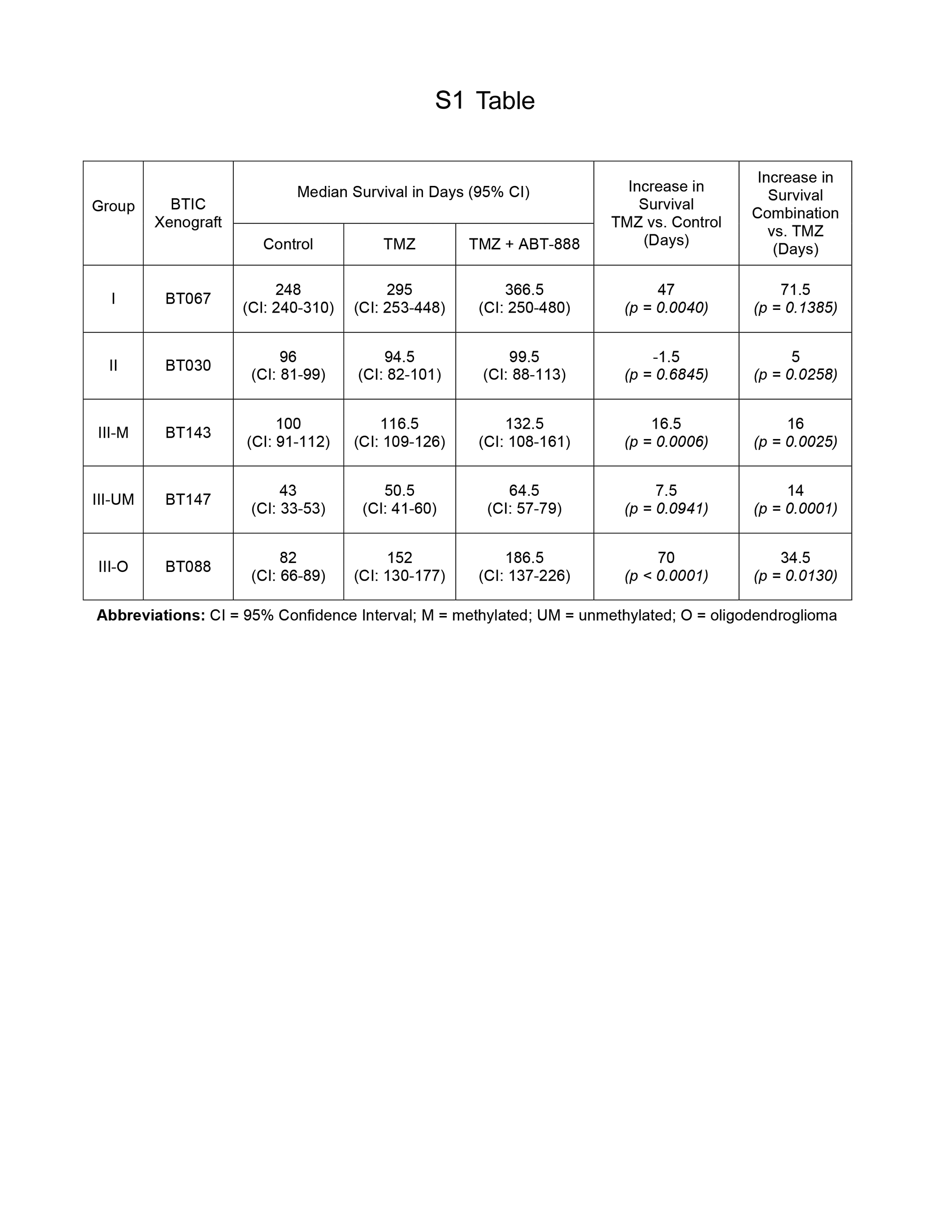

Supplement: S1 Table — (TIF) [file pone.0202860.s004.tif]
